# Protein Modification by Dicarbonyl Molecular Species in Neurodegenerative Diseases

**DOI:** 10.4061/2011/461216

**Published:** 2011-03-14

**Authors:** Wesley M. Williams, Aaron Weinberg, Mark A. Smith

**Affiliations:** ^1^Department of Biological Sciences, Case Western Reserve University, Cleveland, OH 44106, USA; ^2^Department of Pathology, Case Western Reserve University, Cleveland, OH 44106, USA

## Abstract

Neurodegeneration results from abnormalities in cerebral metabolism and energy balance within neurons, astrocytes, microglia, or microvascular endothelial cells of the blood-brain barrier. In Alzheimer's disease, *β*-amyloid is considered the primary contributor to neuropathology and neurodegeneration. It now is believed that certain systemic diseases, such as diabetes mellitus, can contribute to neurodegeneration through the effects of chronic hyperglycemia/insulin resistance resulting in protein glycation, oxidative stress and inflammation within susceptible brain regions. Here, we present an overview of research focusing on the role of protein glycation, oxidative stress, and inflammation in the neurodegenerative process. Of special interest in this paper is the effect of methylglyoxal (MGO), a cytotoxic byproduct of glucose metabolism, elevated in neurodegenerative disease, and diabetes mellitus, on cerebral protein function and oxidative stress. How MGO interacts with amino acid residues within *β*-amyloid, and small peptides within the brain, is also discussed in terms of the affect on protein function.

## 1. Introduction

Approximately 24 million people worldwide suffer the effects of some form of dementia, with numbers expected to increase substantially over the next several decades [1]. Neurodegenerative diseases are highly dynamic, multifactorial pathological conditions the causes of which remain poorly understood. The chronic nature of their development and progression suggests an insidious etiology that may involve multiple synergistic interactions among biochemically unrelated molecular species, and perhaps dysfunctional or overcompensatory metabolic, and immunologic pathways. Numerous studies have contributed to our improved, yet far from complete understanding of the etiological causes of Alzheimer's disease (AD), multiple sclerosis, Pick's disease, Creutzfeldt-Jakob disease (CJD), and Parkinson's disease (PD). Many of these studies suggest several mechanistic similarities among these clinically disparate diseases, including abnormalities in protein folding and aggregation, elevated release of intracellular reactive oxygen species (ROS) leading to development of oxidative stress and inflammation, and increased glycation of key cellular proteins [2]. In this paper we will address key elements of our current level of understanding as they relate to the effects of oxidative stress and inflammation on neuronal degeneration. We will then focus on the abnormal glycation of proteins and small peptides relevant to specific neurodegenerative diseases and how the formation of aldehydic molecular species, such as the *α*-dicarbonyls methylglyoxal (MGO) and glyoxal (GO), contribute to the chronic development of this process. MGO is the most reactive dicarbonyl and *in vivo* during aerobic glycolysis is formed by the spontaneous decomposition of triose phosphate intermediates, although MGO can also form from the oxidative degradation of pentoses, ascorbate, and lipids [3]. Finally, we will discuss the mechanisms by which certain proteins and peptides prove susceptible to nonenzymatic glycation (Maillard reaction) through interaction of these dicarbonyls with specific amino acid residues of their primary structure. This paper is intended to be a succinct overview of current concepts regarding the role of protein glycation in the development of oxidative stress and inflammatory response within the CNS. Perhaps concurrently, posttranslational modification of proteins can also occur via oxidative and lipoperoxidative reaction with susceptible amino acids. These reactions, together with glycoxidation likely contribute by varying degree to neurodegeneration associated with AD and CJD [4, 5].

## 2. Neurodegenerative Diseases-Etiological Similarities

Neurodegenerative disorders of the central nervous system are slowly developing, insidious conditions that contribute to initial neuronal cell degeneration and ultimately cell death. Noteworthy is the occurrence of specific pathological markers that denote the major neurodegenerative diseases, including AD, PD, and CJD. While the cytological location and expression of these posttranslational pathological markers may vary each of these disorders involves (1) abnormal protein folding and aggregation and (2) elevated levels of nonenzymatic protein glycation. Dicarbonyl molecular species (MGO and GO) are unavoidable byproducts of sugar metabolism and are known contributors to the misfolding, aggregation, and insolubility of susceptible proteins through their ability to adduct and form crosslinks via stable advanced glycation end products (AGEs) [6, 7]. Using PrP27-30 an infective variant of prion protein (PrP^C^) isolated from infected Syrian hamster brain [8], Panza et al. [9] showed *in vitro* that AGE formation by GO and MGO is more highly adducted in noninfectious PrP than in infectious PrP27-30. Interestingly, they found AGE-modification of prion resulted in reduced fibril formation, an event that may actually contribute to reduced prion infectivity *in vivo*. In other neurodegenerative diseases, such as AD the effect of AGE-related adduct formation may contribute to modification of both intracellular (lipofuscin, neurofibrillary tangles; NFTs) and extracellular proteins (*β*-amyloid) [10–12]. In addition to their effects on protein folding and aggregation AGEs are likely contributors to the generation of ROS and nitrogen oxygen species, including the radicals superoxide and peroxynitrite in AD and other neurodegenerative diseases [13]. Superoxide (O_2_^∙−^) not only can react with nitric oxide (NO^∙^) to form the reactive nonradical peroxynitrite (ONOO^−^), but also in the presence of transition metals (e.g., Fe^2+^), and Fenton chemistry produces the nonradical hydrogen peroxide (H_2_O_2_) that decomposes to the hydroxyl anion (OH^−^) and highly reactive hydroxyl radical (OH^∙^) [14, 15].

## 3. Alzheimer's Disease-Protein Glycation and **β**-Amyloid

A well-established hallmark of Alzheimer's neuropathology is the excessive presence of extracellular senile plaques, comprised predominantly of *β*-amyloid and NFT [16]. Previous study has shown that the glycation process results in the extracellular formation of adducts and AGEs on *β*-amyloid [17], that MGO is a major glycating agent in this process [18], and that an accelerated polymerization of the peptide occurs as a result of AGE crosslinkage [19]. These findings have led to the proposal that AGEs may act to promote increased *β*-amyloid and plaque deposition within the AD brain [2]. AGEs also adduct to proteins intracellularly, including NFT with the level of glycation increasing with age and severity of AD [20].

In a recent review by Münch and colleagues [2], a sequence of events has been outlined detailing the involvement of increased production of MGO through accumulation of triose phosphates [21]. Triosephosphate isomerase (TPI) is an enzyme that interconverts dihydroxyacetone phosphate (DHAP) and d-glyceraldehyde phosphate [22], thus limiting the metabolic production of MGO under physiological conditions [23, 24]. It is the only glycolytic enzyme that exhibits a functional deficiency correlated with neurodegenerative disease [25]. The deficiency in TPI function results in the accumulation of DHAP that spontaneously degrades to MGO [26]. Dysfunctional TPI in AD appears to result from a *β*-amyloid-dependent nitrosylation of tyrosine in TPI as demonstrated in both AD brain and in mouse brain overexpressing *β*-amyloid and presenilin 1 [27]. Together with increased levels of unchelated transition metals (copper and iron) associated with amyloid plaques, glycated proteins become increasingly oxidized, resulting in the elevated presence of cytotoxic proteins [2, 28].

### 3.1. Glycation and the Onset of Oxidative Stress and Inflammation in Neurodegeneration

 The generation of ROS and their cytotoxic effects are common to the pathophysiology of many neurodegenerative disorders. Noteworthy is the mechanism by which these free radicals and free-radical-producing substrates, such as H_2_O_2_, are generated. Evidence for the presence of oxidative stress within the AD brain is substantial [29–32] and is multifactorial, originating from peroxidation of lipids by aldehydes such as hydroxynonenal [33], protein and DNA oxidation by carbonylation [34], and glycation-related reactions [35] suggesting an imbalance between the abnormal production of free-radical species and the compromised cellular mechanisms responsible for their neutralization. The question arises however, as to whether the onset of oxidative stress is an early feature of neurodegenerative diseases or an epiphenomenon. The answer to this question remains equivocal, although there is considerable evidence to support the involvement of ROS and oxidative stress in the early development and progression of neurodegeneration. In PD, ROS are produced at a higher rate than that observed in age-matched healthy controls. Moreover, appearance of ROS occurs in association with the presence of AGEs in Lewy bodies prior to appearance of the Parkinson's phenotype [36].

The well-characterized nonenzymatic reaction of reducing sugars glucose, fructose and glyceraldehyde with susceptible amino acid residues of proteins can lead to the irreversible formation of the Schiff base, and the more stable Amadori product [37]. The reaction may proceed further by condensation, dehydration, and additional rearrangements to form advanced glycation endproducts or AGEs [38]. In addition to the potential contribution of *β*-amyloid to protein oxidation and oxidative stress [39] experimental evidence supports the underlying involvement of accelerated AGE formation as a significant contributor to oxidative stress and neurodegeneration of the Alzheimer's type [40], not only by the direct production of ROS but also through their binding to the multiligand receptor RAGE, a cell-surface molecule and member of the immunoglobulin superfamily [41, 42]. In both *in vitro* and *in vivo* studies Ko and colleagues [43] show that AGEs up-regulated the expression of APP, a response that could be prevented by the pretreatment of neuroblastoma cells (cell line SH-SY5Y) with the ROS inhibitor N-acetyl-L-cysteine. Furthermore, the accumulation of *β*-amyloid_1–42_ was also increased following the treatment of these cells with AGE.

The reciprocal interaction between oxidative stress, through protein oxidation and lipid peroxidation, and the activation of proinflammatory pathways as part of the innate immune response within the brain has been extensively studied and shown to be a biologically significant component of neuropathogenesis in AD [43, 44]. This was demonstrated histochemically in AD brain, where activated microglial cells and astrocytes, both resident immune cells of the brain [45], express proinflammatory cytokines, including interleukin (IL)-1*β*, IL-6, and tumor necrosis factor-*α*, along with ROS [46]. This expression of proinflammatory cytokines may result from the binding of *β*-amyloid_1–42_ fibrils to complement factor C1q and serum amyloid P component, as shown by Eikelenboom et al. [47] in cultures of human microglial cells. The AGE-RAGE pathway is a significant contributor to this process in part through the ability of RAGE to bind with various proinflammatory ligands, including AGEs and *β*-amyloid and through the generation of ROS as second messengers [48]. This was demonstrated by Gasic-Milenkovic et al. [49] who showed the inflammatory response from murine microglial cells was augmented by exposure to AGE-bound *β*-amyloid, relative to unbound *β*-amyloid. Neuroinflammation and impairment of neurogenesis in AD may be promoted not only by *β*-amyloid, but also by the intracellular domain of expressed amyloid precursor protein (AICD), as shown by Ghosal et al. [50] in transgenic mice coexpressing the 59-residue long AICD fragment and in APP-knockout mice.

### 3.2. MGO Cytotoxicity and Neurodegeneration

MGO is the most reactive *α*-dicarbonyl and is increased in normal aging and especially in neurodegenerative disease [51]. Exposure of neuroblastoma cells to MGO for up to 24 hrs induces depolarization of the plasma membrane, release of glutamate, and formation of ROS [52]. Experimental evidence also confirms the capability of MGO to impair cellular glucose metabolism and energy depletion, as shown in cultured neuroblastoma cells [53]. Elevated MGO in Type 2 diabetes and in at least some neurodegenerative diseases, including AD strongly supports the proposal that dicarbonyl stress, particularly that due to MGO contributes significantly to alterations in protein function in these conditions and to their neuropathology. Support for this proposal comes from experiments on cultured rat hippocampal neuronal cells exposed to MGO [54]. MGO induced oxidative-stress related neuronal cell death and concurrently induced expression of nerve growth factor and release of proinflammatory cytokine IL-1*β*. Studies also demonstrate the ability of MGO to inhibit protein function. Argpyrimidine, formed by the reaction of MGO with arginine residues, is a specific marker for protein glycation and is present in human renal tissue and lens proteins from diabetic patients, as well as in at least some neurodegenerative conditions [55–57]. These results lend additional support for a central role of dicarbonyl molecular species in vivo, especially MGO, in the early development and progression of oxidative stress and inflammation in diabetes and neurodegenerative disease.

## 4. Diabetes Mellitus—A Risk Factor for AD?

Considerable evidence is presented in the literature supporting the association of the 2 major components of Type 2 diabetes mellitus, insulin resistance and hyperglycemia with dementia of the Alzheimer's type [58, 59], although acceptance of this association is not universal. An often referenced longitudinal study supporting an association is the Rotterdam study reported by Ott et al. [60]. Study data gathered from 5,000 subjects 55 years of age and older suggested a strong correlation between Type 2 diabetes and the subsequent development of AD. Baker et al. [61], reporting on a study of human subjects diagnosed with diabetes or presenting with elevated glycemic indices showed that individuals with increased insulin resistance also presented with an AD-like pattern of reduced cerebral glucose metabolic rate in frontal, parietotemporal, and cingulated regions of the brain, suggesting insulin resistance as a predictive marker for AD. Additional support for Type 2 diabetes as a risk factor for AD comes from studies by Curb et al. [62] and Leibson et al. [63], among others. In view of recent reports, a causative association between Type 2 diabetes, perhaps as a result of hyperinsulinemia and/or hyperglycemia, and cognitive impairment is strong, with an association to AD somewhat less so. Although both diseases exhibit some similarities, such as insulin resistance [64], dysregulation of iron homeostasis, and increases in metallothionein and heme oxygenase-1 [65], there is limited evidence that Type 1 or Type 2 diabetes leads to AD neuropathology [66]. 

Overall, study findings to date suggest a strong correlation between chronic Type 2 diabetes and subsequent development of dementia, however, the effect of diabetes on cerebral function may be related more to an initial predisposition to vascular dementia than to development of classical AD neuropathology [67, 68]. In the following section we will discuss the involvement of insulin resistance and hyperglycemia, both hallmarks of Type 2 diabetes in the pathophysiology of neurodegeneration, including AD.

## 5. Diabetes Mellitus Type 2 and Neurodegeneration

Although some question remains as to the predisposition of the diabetic brain to develop AD pathology, recent studies suggest a strong correlation between Type 2 diabetes, brain atrophy and reduced neuronal integrity. In one clinical study [69] of 89 patients with diabetes, 70 to 82 years of age MRI scans and cognitive function testing was conducted at baseline and again 3 years later. After correcting for age, gender, educational level, hypertension, and treatment with pravastatin linear regression analysis revealed statistically significant atrophy of the brain in diabetic patients in comparison to matched control subjects without diabetes. A second MRI study by de Bresser et al. [70] conducted over 4 years on 55 patients with Type 2 diabetes showed significant reduction in total brain volume and increased peripheral cerebrospinal fluid volume, leading to the conclusion that Type 2 diabetes can lead to slow, chronic cerebral atrophy. Encephalopathy with neuronal degeneration and inflammation has also been demonstrated in the hippocampus of the streptozotocin (STZ) Type 2 diabetic rat model [71]. The hyperglycemia associated with Type 2 diabetes also appears to impair gap junctional communication between astrocytes as shown in (1) cultured astrocytes harvested from 1-day-old Wistar-Hanover rats and exposed to high glucose, and (2) brain slices from 20-to-24-week-old STZ rats [72]. Assessment of gap junction channel-mediated dye transfer was used to determine astrocytic gap junctional function. 

Difficulty in determining the causative relationship between Type 2 diabetes and AD is, in part, due to the heterogeneous nature of AD. A definitive diagnosis of AD can only be made postmortem. Moreover, AD is frequently associated with a microvascular component [73], and as Luchsinger and Gustafson correctly state [74], AD may illustrate a neurodegenerative disease with a graded overlapping of vascular and amyloid neuropathologies. 

The AD brain exhibits regional glucose hypometabolism as well as abnormalities in insulin signaling [75], both components of diabetes mellitus [76, 77]. The presence of glucose hypometabolism and development of a hyperglycemic cellular microenvironment precipitated by progressive insulin resistance and altered insulin signaling pathways contribute to neuronal death [78]. MGO may promote neuronal cell death by contributing directly to insulin resistance via acceleration of AGE formation [79], and also by involvement in the combined effects of dicarbonyl reactivity with tau, and hyperphosphorylation of tau and other proteins by protein O-glycosylation via monosaccharide *β*-N-acetylglucosamine attachment to serine/threonine residues via an O-linked glycosidic bond [80]. Present data suggests that glucose hypometabolism and insulin resistance in AD contribute to the development of tau hyperphosphorylation by reduction of this glycosylation process, thus leading to the formation of tangles and eventual neuronal cell death [81]. Increased tau phosphorylation has also been described in a mouse model of Type 1 (STZ injected) and 2 (db/db) diabetes mellitus [82]. In this study, tau phosphorylation was found to increase in the cortex and hippocampus of both mouse models, but the increase was particularly noteworthy in the STZ-injected mouse brain. Study results suggested that tau phosphorylation was directly induced by hyperglycemic (Type 2) and insulin deficiency (Type 1). Similar results were reported by Ke et al. [83] in the pR5 transgenic mouse model of AD with superimposition of Type 2 diabetes induced by STZ. Immunocytochemical and Western blot analyses indicated tau hyperphosphorylation was accelerated in the diabetic transgenic model. Hyperglycemic conditions in Type 2 diabetes and perhaps in AD can elevate tissue dicarbonyl levels, primarily MGO and GO molecular species, thus contributing to tau crosslinking and oligomer formation. That this can occur has been shown by Kuhla et al. [84], who demonstrated increased dimer and oligomer formation in wild-type and pseudophosphorylated mutant tau proteins, particularly when exposed to MGO.

## 6. Dicarbonyl Interaction with Proteins—Amino Acid Residues and Protein Susceptibility

Production of *α*-dicarbonyl molecular species, most notably GO and MGO, occurs slowly under physiological conditions, yet adduction to susceptible lysine, arginine and cysteine residues occurs, with the hydroimidazolones being the most commonly formed adducts, primarily on proteins with long half-lives [85, 86]. Compromise of protein function is effected initially by glycation of the lysine side chain and N-terminal amino acid residues, followed by later stage modification involving not only these, but arginine and cysteine residues and stabilization to AGEs via Schiff base and Amadori rearrangement [87, 88]. The impact of glycation on protein function depends in part on the turnover rate of the protein, and cellular defense mechanisms, such as activity of the glyoxalase system responsible for the degradation of unadducted dicarbonyl [89]. Because activity of the glyoxalase system declines with progression of Type 2 diabetes and AD [84] MGO levels can rise significantly. Glyoxalase activity depends on the availability of reduced glutathione which can be decreased in neurodegenerative disease [90].

Both *in vitro* and *in vivo *studies indicate that presence of glycating agents, such as MGO, within the vicinity of arginine, lysine, and cysteine residues of a protein is not necessarily predictive of adduction at these sites. Moreover, glycation and reactivity of dicarbonyls with amino acids is a dynamic process. Jia et al. [91] demonstrated *in vitro* the susceptibility of insulin to glycation by MGO, while glucagon, a protein of similar mass with two arginines and a single lysine, was resistant to modification. Additional support for the selective nonenzymatic glycation of proteins comes from studies of human [92], mouse, and rat [93] plasma proteins obtained from aged populations. Both studies show glycation may be limited to relatively few proteins.

The tertiary structure of a protein, and therefore accessibility of the glycating agent to susceptible residues, is a primary determinant of whether the glycation process proceeds. This was shown by Gao and Wang [94] who correlated selective modification of specific arginine residues within the hemoglobin molecule with solvent accessibility, as determined by the “relative surface exposable area” calculated for each arginine within the native molecule. Conversely, in an earlier study on glycation of RNase protein Watkins et al. [95] note that the only common characteristic of unreactive lysine residues in the protein were their location on the protein surface, and thus readily solvent accessible. They point out, however, that this nonreactivity may be due to a calculated 20% reduction in the rate constant for glycation following reaction of highly reactive lysine residues. A second determinant of site specificity of protein glycation is the p*K*_*A*_ of the competing residues, with the residue having the lower value more susceptible to initial Schiff base formation and subsequent conversion to stable glucosamines via the Amadori rearrangement [96]. However, the initial formation of the Schiff base is not necessarily predictive of the Amadori rearrangement, as shown by the observation that Lys-41 in bovine pancreatic RNase A although less susceptible to Schiff base modification than is *N* ^*α*^-lys-1, accounts for more than twice the Amadori adduct to the RNase. It should be emphasized that failure of the amino acid to initially react with the glycating agent does not preclude eventual modification. Thirdly, reactivity with glucose appears to be enhanced when specific residues, like lysine, are proximal to high-affinity binding sites for phosphate ion or organic phosphate often within basic regions of the molecule, a frequent condition within substrate and effector binding sites of the protein [95]. Proximity of lysine to carboxylic acid containing residues within the primary or tertiary structure has been noted by Shapiro et al. [97].

## 7. Discussion and Summary

The preponderance of reported studies support an association between hyperglycemia and insulin resistance/hyperinsulinemia, both components of uncontrolled Type 2 diabetes, and similar conditions in AD. Increased glycation of susceptible proteins is also of pathophysiological significance in both diabetes and neurodegenerative diseases. Attempting to demonstrate Type 2 diabetes as a risk factor for AD has been inherently difficult for 2 reasons: (1) the percentage of study subjects maintaining good glycemic control has often not been strictly recorded, thus these individuals may present with few, if any systemic complications such as cardiovascular, renal, or peripheral nerve pathology most often associated with diabetes and (2) confirmation of AD cannot be made until postmortem examination of brain sections. To date, there are no reported study findings relating the level of long-term glycemic control to cognitive function in Type 2 diabetes. However, in 2007, Williamson et al. [98] initiated the Action to Control Cardiovascular Risk in Diabetes Memory in Type 2 Diabetes Study (ACCORD-MIND), the first such study to formally assess cognitive outcome following a treatment program designed to compare the effects of standard care versus intensive care guidelines. This study should contribute importantly to our understanding of how, and whether long-term stability of blood sugar affects cognitive function. 

The role of protein and peptide glycation, and that of MGO and other dicarbonyl molecular species, in the development and progression of neurodegenerative diseases remains poorly defined, due largely to a lack of rigorous corroborative studys conducted* in vivo*. Interpretation of *in vitro* findings are often tentative, given that tissue localization, concentration, half-life, and reactivity of key reactants, that is, dicarbonyl molecular species, susceptible protein, and host defense systems, such as antioxidants, reduced glutathione, and glyoxylase I and 2 may not be reflective of disease conditions *in vivo*. Nonetheless, there is considerable evidence supporting the role of protein glycation and associated oxidative stress and inflammation as central to the progression of some neurodegenerative diseases, AD being a prime example [99]. However, whether these events are early, causative events in the disease remains unclear, although Smith et al. [99] point out that the presence of AGE epitopes localized to diffuse *β*-amyloid plaques represent an early pathological event in AD. Oxidative stress brought about by glycation of amino acids, and the irreversible formation of AGEs is likely to be but one of several pathways contributing to protein dysfunction. Recently, elevation of redox-active iron was found in the cerebral cortex and cerebellum in human preclinical AD brains, and brains with mild cognitive impairment (MCI), indicating transition-metal-induced free radical generation within these brain regions [100]. Occurrence of increased iron in brain from MCI patients is also highly suggestive of an iron-generated free radical-induced primary event within this and perhaps other neurodegenerative diseases that may respond to the use of antioxidant therapies. Elevated levels of transition metals within AD brain may be primary contributors to the augmentation of oxidative stress associated with amino acid-MGO adduct formation. Concurrently, MGO-induced AGE formation and AGE-RAGE binding can regulate expression of proinflammatory cytokines via redox-sensitive pathways that may be central to neuroinflammation in AD [44]. Potential therapies for AD and other neurodegenerative diseases focusing on antioxidants, anti-inflammatory agents, and inhibitors of AGE-RAGE pathways are currently intense areas of research. 

In recent years an increasing number of animal models have been developed that closely approximate conditions reflecting the human disease. Increased emphasis on utilization of these *in vivo* models in long-term studies of dicarbonyl-protein interactions is a plausible and necessary alternative for future investigations. Increased focus on *in vivo* studies will be required to determine, conclusively, the relative involvement of dicarbonyl-induced glycation, oxidative stress, and inflammatory responses in the etiology of human neurodegenerative disease.
